# Cyclosporin A Inhibits the Influenza Virus Replication through Cyclophilin A-Dependent and -Independent Pathways

**DOI:** 10.1371/journal.pone.0037277

**Published:** 2012-05-15

**Authors:** Xiaoling Liu, Zhendong Zhao, Zheng Li, Chongfeng Xu, Lei Sun, Jilong Chen, Wenjun Liu

**Affiliations:** 1 Center for Molecular Virology, Key Laboratory of Pathogenic Microbiology and Immunology, Institute of Microbiology, Chinese Academy of Sciences, Beijing, China; 2 Graduate University of Chinese Academy of Sciences, Beijing, China; 3 China-Japan Joint Laboratory of Molecular Immunology and Molecular Microbiology, Institute of Microbiology, Chinese Academy of Sciences, Beijing, China; Scripps Research Institute, United States of America

## Abstract

The immunosuppressive drug cyclosporin A (CsA) has inhibitory effects on the replication of several viruses. The antiviral effects are through targeting the interaction between viral proteins and host factor cyclophilin A (CypA). CypA has been identified to interact with influenza A virus M1 protein and impair the early stage of the viral life cycle. In order to identify the effect of CsA on influenza virus replication, a CypA-depleted 293T cell line, which was named as 293T/CypA−, was constructed. The cytopathic effect (CPE) assay and the growth curve results indicated that CsA specifically suppressed the influenza A virus replication in a dose-dependent manner. CsA treatment had no effect on the viral genome replication and transcription but selectively suppressed the viral proteins expression. Further studies indicated that CsA could impair the nuclear export of viral mRNA in the absence of CypA. In addition, the antiviral activity of CsA was independent of calcineurin signaling. Finally, CsA could enhance the binding between CypA and M1. The above results suggested that CsA inhibited the replication of influenza A virus through CypA-dependent and -independent pathways.

## Introduction

Many of interactions between influenza viral components and host factors have now been identified. Emerging data indicate that their identification and characterization will provide new insights into the mechanisms by which viruses complete their life cycle. Furthermore, such knowledge would illuminate potentially useful targets for therapeutic intervention. For example, human immunodeficiency virus type 1 (HIV-1) has been tested or treated with the antiviral drugs targeting host cell factors involved in viral replication [1]. However, this goal would generally take several decades to achieve with conventional genetic screening methods and mammalian cell cultures.

The well-known immunosuppressive drug cyclosporin A (CsA) is a cyclic 11-amino-acid peptide produced by the fungus *Tolypocladium inflatum*. It was reported that CsA had antiviral activity on the replication of several viruses through targeting the interaction between the viral proteins and host factor CypA, which is the major intracellular receptor for CsA [2], [3], [4], [5], [6]. For example, CsA can disrupt the interaction of Gag-CypA in vitro, block CypA incorporation into virions, and inhibit viral replication [4], [5]. CsA inhibits hepatitis C virus replication mainly through CypA [3], [7], [8], [9]. In addition, it has been reported if administered with a dose of influenza virus lethal for normal mice, CsA-treated mice greatly survived compared to control, suggesting CsA may inhibit the influenza virus replication [10]. In the previous study, CypA has been identified to interact with influenza A virus M1 protein and accelerate the degradation of M1 protein [11], [12]. Therefore, it is of interest to investigate the effect of CsA on influenza A virus replication at the cell level and to determine in more detail whether the regulation of influenza viral replication by CsA involves the CypA interaction with M1.

In the present study, we investigated the effect of CsA on the intracellular replication of influenza A virus, using a control cell line which was named as 293T/CypA+ as control and a CypA depleted 293T cell line which was named as 293T/CypA−. The results indicated that CsA inhibited the replication of influenza A virus at the post transcription level. The molecular mechanism of CsA was not only through CypA-dependent pathway but also CypA-independent pathway.

## Results

### CsA inhibited the replication of influenza A virus in a dose-dependent manner

**Figure 1:**
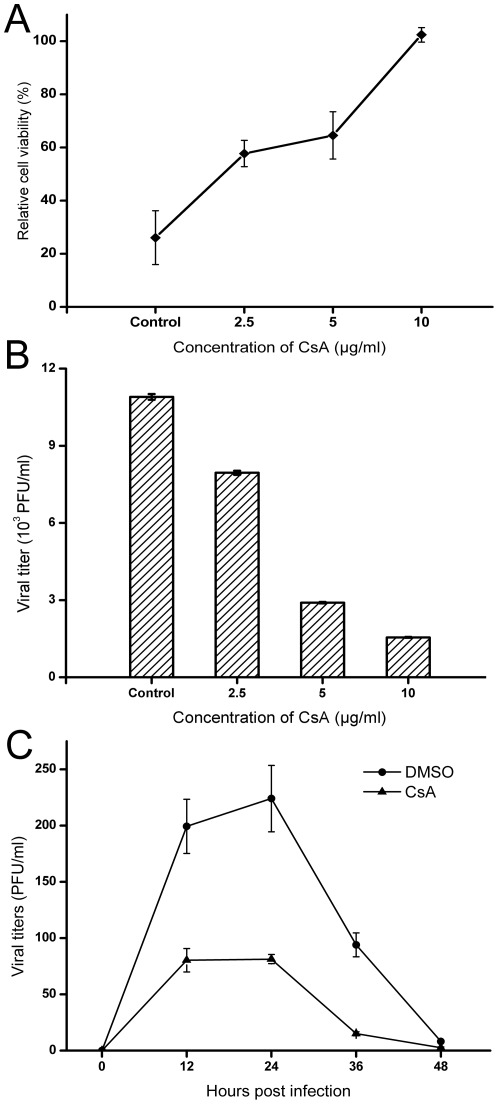
CsA inhibited the influenza virus replication in MDCK cell line. A: Cytopathic effect assay of CsA on the influenza virus replication. Monolayers of MDCK cells on 96-well microwell plates were infected with influenza A/WSN/33 (MOI = 0.1). Infected cells were treated in duplicate with different concentrations of CsA (Control, 2.5 µg/ml, 5 µg/ml, 10 µg/ml) in DMEM with 2 µg/ml TPCK-treated trypsin, and the preparations were incubated at 37°C for another 36 h, after which the monolayers were stained with crystal violet (0.1% in 20% ethanol) and examined for cytopathic effect (CPE). Data are presented as the mean plusminus standard deviation (± SD) from three independent experiments. B: Plaque assay of the titer of viruses in the supernatant with different concentrations of CsA (0–10 µg/ml). Infected cells were treated in duplicate with different concentrations of CsA (0 µg/ml, 2.5 µg/ml, 5 µg/ml, 10 µg/ml) in DMEM with 2 µg/ml trypsin, and the preparations were incubated at 37°C for another 16 h, after which the titer of viruses in the supernatant was analyzed by plaque assay. Data are means ± SD of three separate experiments. C: The growth curves of influenza virus in MDCK cell line in the absence or presence of CsA (5 µg/ml).The cells were infected with influenza A/WSN/33 (MOI = 0.01) for 1 h. After being washed with PBS 3 times, the cells were cultured with fresh medium supplemented without or with CsA (5 µg/ml). At various times post-infection, viral titers in the supernatants were determined by plaque assay. Data are means ± SD of three separate experiments.

The effect of CsA on influenza A virus replication was investigated at the cell level. A dose response curve with various concentrations of CsA (control, 2.5–10 µg/ml) revealed that, at 36 h p.i., less cytopathic effect (CPE) observed in the higher treated concentration of CsA and more CPE in the lower treated concentration of CsA in MDCK cells infected with influenza virus of H1N1 subtype (A/WSN/33) (Figure 1A). In addition, CsA had similar effects on the influenza virus of H9N2 subtype (A/Chicken/Liaoning/1/00) infected MDCK cells (data not shown). The present data showed that CsA effectively inhibited the influenza A virus when CsA was added to the cell culture following virus adsorption. At 16 h p.i., the titer of viruses in the supernatant was analyzed by plaque assay. As shown in Figure 1B, the titer of viruses in the supernatant was decreased with the increasing concentrations of CsA. No significant cytotoxic effects were observed in uninfected cells exposed to 2.5–10 µg/ml of CsA. Since 10 µg/ml of CsA caused marked morphological alterations and decreased cell viability (data not shown), all subsequent experiments were performed with 5 µg/ml of CsA.

The multiplication assay was performed to determine the antiviral effect of CsA. MDCK cells were infected with influenza A/WSN/33 (MOI = 0.01) for 1 h. After being washed with phosphate buffered saline (PBS) three times, the cells were cultured with fresh medium supplemented with or without 5 µg/ml of CsA. At various time points post infection, viral titers in the supernatants were detected by plaque assay. As shown in Figure 1C, the growth curve indicated that CsA inhibited the influenza virus replication in MDCK cells.

### CypA inhibited the replication of influenza virus in CypA rescued cells

**Figure 2:**
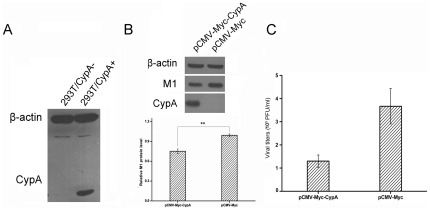
CypA inhibits the replication of influenza virus in CypA rescued cells. A: Western blot assay of the amount of CypA in both 293T/CypA+ and 293T/CypA− cell lines. B: Re-expression of CypA in 293T/CypA− cell line. 293T/CypA− cells were transfected with plasmids (4 µg) encoding Myc-tagged WT CypA or pCMV-Myc as control. At 36 h p.t., transfected cells were infected with WSN virus (MOI = 0.1). At 16 h p. i., total cell lysates were detected by Western blot using anti-M1, anti-Myc and anti-β-actin. The protein levels of M1 were quantified. Data are presented as ± SD from three independent experiments. Significant differences (P<0.01, t-test) are indicated by two asterisks. C: Re-expression of CypA in 293T/CypA− cell line inhibited influenza virus replication. The media were collected and assayed for virus titers by plaque assay. The data represent the means of three independent experiments.

In order to study the mechanism of CsA in the influenza A virus replication, the CypA-depleted stable cell line and the luciferase-depleted cell line as control were established using the pSUPER RNAi System [12]. Several clonal populations of GFP expressing cells were obtained and measured for CypA depletion. The amount of CypA in cell was characterized. There was no detectable CypA in 293T/CypA− cell line compared with that in 293T/CypA+ cell line. The amount of β-actin as control was similar in both cell lines as shown in Figure 2A. In addition, except for the expression level of CypA, no significant differences in cell morphology and cell cycle were observed between the two cell lines. When CypA was rescued through transfecting with pCMV-Myc-CypA in 293T/CypA− cell line, the intracellular expression level of M1 in CypA-transfected cells was lower than the vector control (Figure 2B). Plaque assay showed that at 16 h p.i., the number of virus particles that were released from CypA-transfected 293T/CypA− cells resulted in nearly three-fold reduction of the virus titer levels seen in the cells transfected with a pCMV-Myc empty plasmid (Figure 2C).

### CsA inhibited the viral replication through cyclophilin A-dependent and -independent pathways

**Figure 3:**
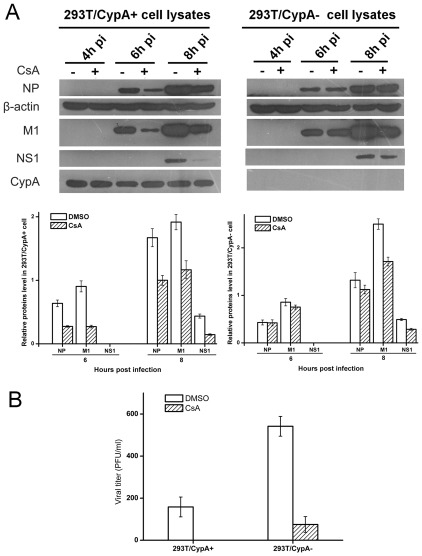
CsA selectively inhibited the viral proteins levels in both 293T/CypA+ and 293T/CypA− cells. Both cell lines were infected with inﬂuenza A/WSN/33 at MOI 1. After being washed with PBS, the cells were cultured with fresh medium supplemented without or with CsA (5 µg/ml). At 4 h p.i., 6 h p.i. and 8 h p.i., the cell lysates were collected to detect the M1, NP and NS1 proteins. β-actin as an internal control was also detected by Western blot analysis (A). The relative viral protein levels of M1, NP and NS1 in both 293T/CypA+ and 293T/CypA− cells were calculated by quantifying the results shown in panel (A). The viral titers were detected at 8 h p.i. in two cell lines (B). Data are means ± SD of three separate experiments.

To determine whether CsA's inhibition of viral replication was related to the host factor CypA, both 293T/CypA+ and 293T/CypA− cell lines were treated with CsA (5 µg/ml) immediately after influenza A/WSN/33 infection (MOI = 1), and a time-course experiment for antiviral activity of CsA was performed at 4 h, 6 h and 8 h p.i. by Western blot analysis. As shown in Figure 3A, CsA treatment significantly decreased the expression of the M1 protein in both cell lines at 6 h p.i. and 8 h p.i. compared with the control groups. Similar results were obtained in the NS1 protein. While CsA had different effects on the expression of NP protein in two cell lines. In 293T/CypA+ cell line, CsA decreased the expression of NP protein. However, the expression level of NP was little less in the presence or absence of CsA in 293T/CypA− cell line. These interesting results indicated that CsA had two pathways to inhibit the influenza virus replication. One was CypA-dependent pathway. The other was CypA-independent pathway. In addition, we analyzed the viral titers in the supernatants at 8 h p.i. in detail (Figure 3B) when the first viral life cycle was over. In 293T/CypA+ cell line, the viral titer was 158 PFU/ml in the presence of DMSO, while the virus released from the cells in the presence of CsA was too little to be detected at 8 h p.i. In 293T/CypA− cell line, the viral titer was 541 PFU/ml with the treatment of DMSO and it was more than 3 fold of that in 293T/CypA+ cell line. The virus could also be detected in the presence of CsA and the viral titer was 75 PFU/ml. The virus released earlier in 293T/CypA− than in 293T/CypA+ cell line. The results further proved our previous results that CypA was an inhibition factor for influenza virus [11], [12]. Furthermore, CsA inhibited the viral replication through CypA-dependent and -independent pathways.

### CsA inhibited the influenza virus replication through targeting a post-transcriptional level

**Figure 4:**
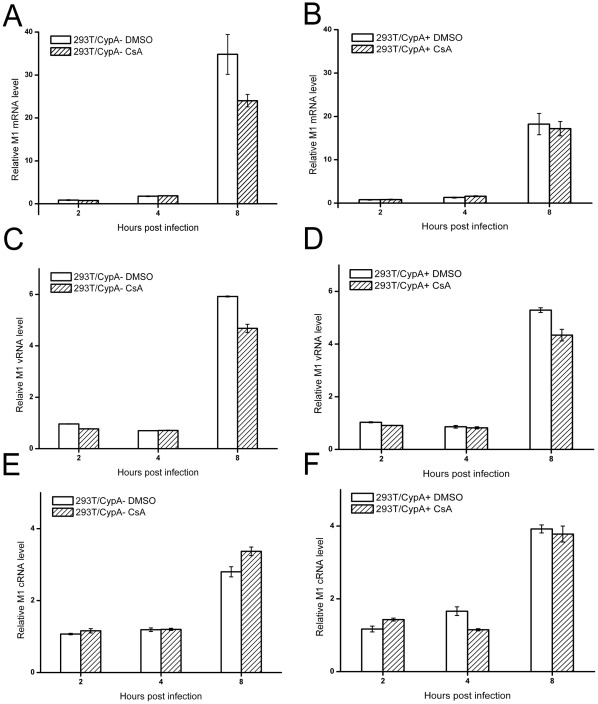
CsA inhibited the influenza virus replication at post-transcriptional level. 293T/CypA− and 293T/CypA+ cell lines were infected with influenza A/WSN/33 (MOI = 1) in the absence or presence of CsA (5 µg/ml). RNA of the infected cells at 2 h, 4 h and 8 h p.i. was extracted and assayed using the specific primers for influenza RNA by RT-PCR. The M1 mRNA level (A and B), vRNA level (C and D) and cRNA level (E and F) of the both cell lines were quantified by real-time PCR analysis using the specific primer for M1. The mRNA of GAPDH was kept as an internal control. Data are means ± SD of three separate experiments.

To understand whether the effect of CsA on the M1 protein expression was related with the transcription or replication phase of the virus life cycle, mRNA, vRNA, cRNA for M1 were measured in control and CsA-treated cells by real-time PCR using specific primers for viral mRNA, vRNA, cRNA at 2 h, 4 h and 8 h p.i. Real-time PCR assays indicated that the M1 mRNA levels were similar in control and CsA-treated cells (Figure 4A, B). Furthermore, there were no significant differences at the levels of M1 vRNA, cRNA in control or CsA-treated cells (Figure 4C to F). Combining with the results that the expression of M1 protein and virus production were significantly decreased with the treatment of CsA, the inhibitory effect of CsA on the M1 protein expression occurred at a post-transcriptional level.

### CsA impaired the nuclear export of viral mRNA in the absence of CypA

**Figure 5:**
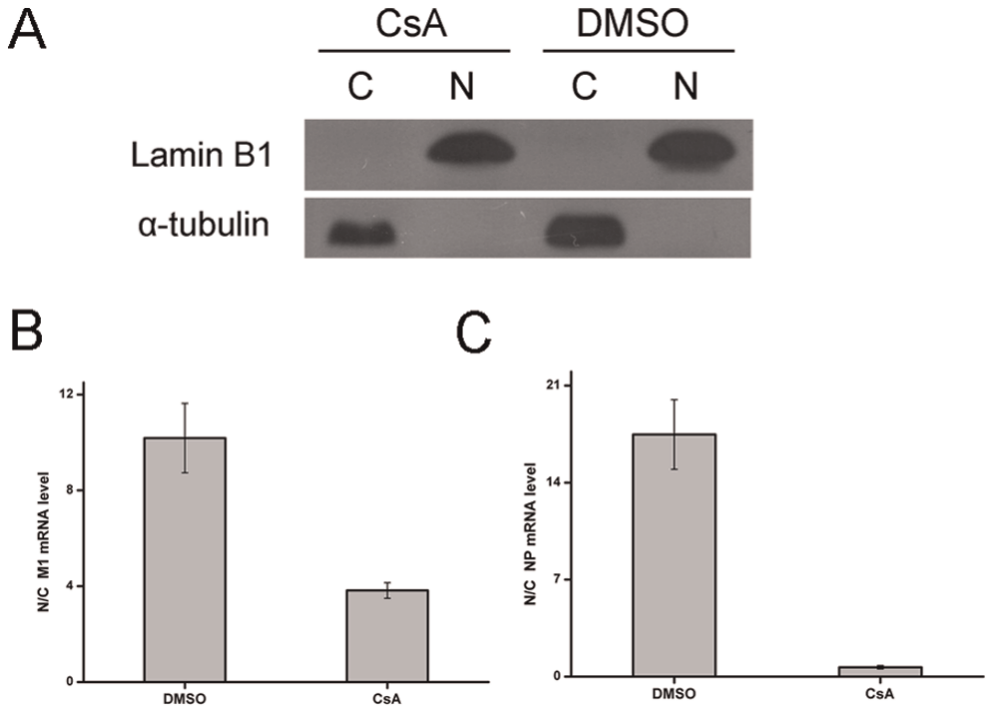
CsA impaired the nuclear export of influenza virus mRNA in the absence of CypA. The 293T/CypA− cell line was infected with A/WSN/33 (MOI = 0.1) in the presence or the absence of CsA. At 4 h post-infection, Nuclear (N) and cytoplasmic (C) components were isolated from the infected 293T/CypA− cell line. One tenth of the components were used for Western blot by anti-alpha-tubulin and anti-lamin B1 (A). The rest of the components were used for the RNA extraction and real time PCR. M1 (B)and NP (C) mRNA levels were quantified by real-time RT-PCR using gene-specific primers. Data are means ± SD of three separate experiments.

As was shown, CsA did not affect the transcription and genome replication of influenza virus. To determine if CsA has an effect on viral mRNA nuclear export, we compared the nuclear and cytoplasmic abundance of M1 and NP mRNA upon influenza virus infection at 4 h post-infection in 293T/CypA− cell line, in which the effect of CypA could be eliminated. First of all, the nuclear and cytoplasmic fractions were isolated and analyzed by Western blotting for lamin B1 and α-tubulin (Figure 5A). The results showed that the nuclear/cytoplasmic fractions were isolated well. With the help of quantitative real time PCR, the mRNA of M1 and NP were detected. These results indicated that the ratio of nuclear/cytoplasm M1 mRNA was lower in CsA treated 293T/CypA− cell line than that in DMSO treated cells (Figure 5B). In addition, we obtained similar results for the pattern of NP mRNA (Figure 5C). These results suggested that CsA could impair the nuclear export of viral mRNA through CypA-independent pathway.

### The effect of CsA on influenza virus replication was independent of calcineurin signaling

**Figure 6:**
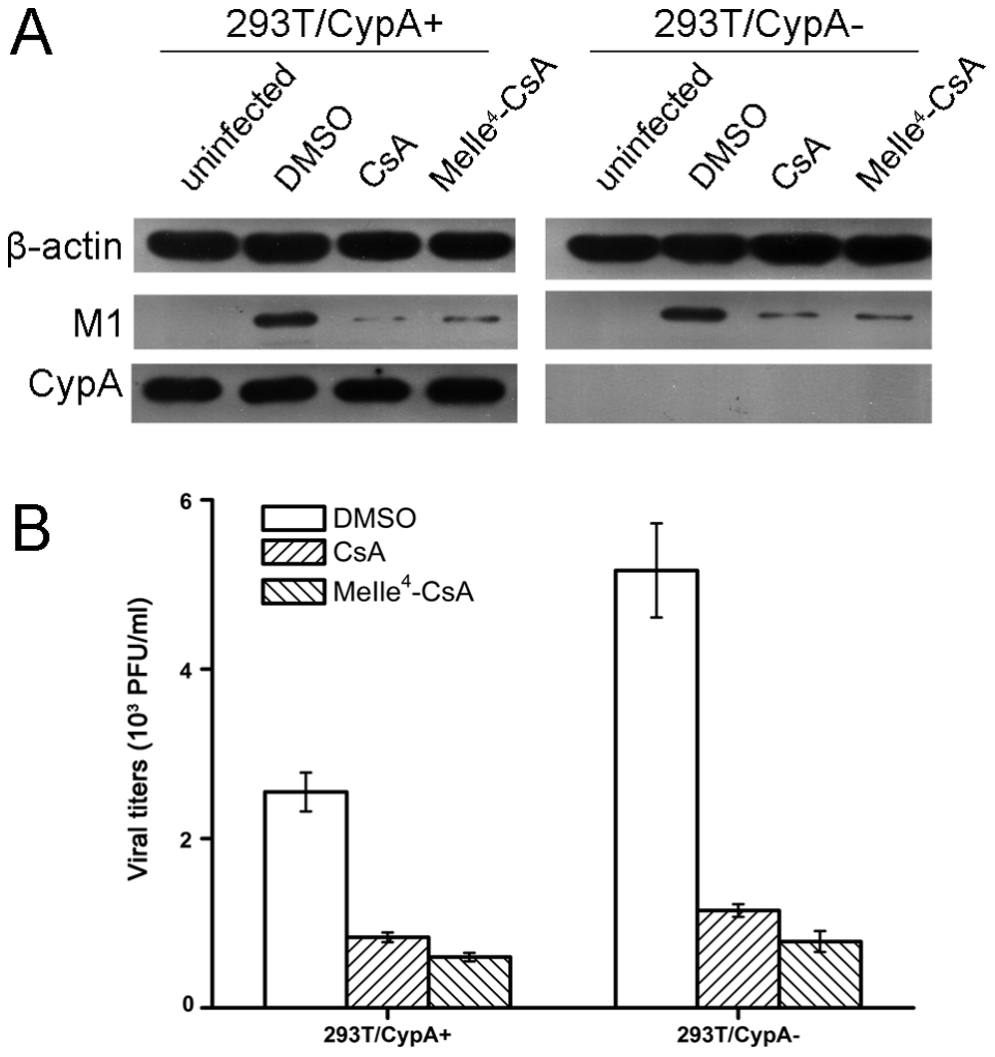
The effect of CsA on influenza virus replication in both 293T/CypA+ and 293T/CypA− cells was independent of calcineurin signaling. Both cell lines were infected with inﬂuenza A/WSN/33 at MOI 1. After being washed with PBS, the cells were cultured with fresh medium supplemented without or with CsA, Melle^4^-CsA (5 µg/ml). At 8 h p.i., the cell lysates were collected to detect the M1 protein. β-actin as an internal control was also detected by Western blot analysis (A). The viral titers in the supernatants were determined by plaque assay (B). Data are means ± SD of three separate experiments.

In mammals, the CsA-CypA complex binds to and inhibits calcium-dependent phosphatase calcineurin [13], [14]. MeIle^4^-CsA is a CsA analogue that binds CypA as tightly as the CsA-CypA complex but does not form a complex with calcineurin. So MeIle^4^-CsA is a nonimmunosuppressive CsA analogue [4], [15]. To identify the mechanism of CsA on the influenza virus replication, 293T/CypA+ and 293T/CypA− cell lines were infected with influenza A/WSN/33 (MOI = 0.1) in the presence or absence of CsA or Melle^4^-CsA, respectively. At 8 h p.i., M1 protein levels in the cell lysates were detected. The results indicated that there was no difference between Melle^4^-CsA and CsA on the expression of M1 protein (Figure 6A). In addition, the viral titer in the supernatant of the CsA treated cells was similar with that of MeIle^4^-CsA treated cells (Figure 6B). Thus, the inhibition of the viral replication by CsA was independent of calcineurin signaling.

### CsA enhanced the binding between CypA and M1

**Figure 7:**
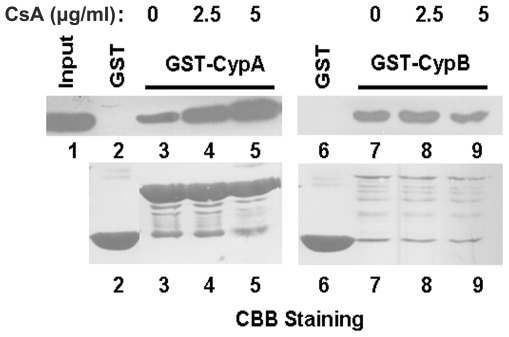
CsA enhanced the binding between CypA and M1. GST pull-down assays between GST-CypA and M1, GST-CypB and M1 were performed in the absence (lane 3, lane 7) or presence (lanes 4, 5, 8, 9) of CsA. The concentrations of CsA in lanes 4, 5, 8, 9 are 2.5, 5, 2.5, 5 µg/ml, respectively. Coomasie brilliant blue (CBB) staining patterns for the pulled-down proteins are shown in the bottom panel.

In the previous study, we found that CypA interacted with M1 and affected the early stage of influenza virus replication. As is shown, CypA, in particular, is the major intracellular receptor for CsA [2]. In order to identify the effect of CsA on the interaction between CypA and M1, GST pull-down assay was performed to demonstrate that the binding capacity between CypA and M1 was increased in a dose-dependent manner with treatment of CsA (Figure 7, lanes 3–5). As a control, we chose another member of cyclophilin family, Cypclophilin B (CypB). CypB could interact with M1 in vitro, the interaction between M1 and CypB was not affected by CsA treatment (Figure 7, lanes 7–9). In our previous study, over-expression or knock-out of CypB has no effect on the influenza virus replication [11]. These results suggested that CsA might be involved in influenza A virus replication partially through regulating the interaction between CypA and M1.

## Discussion

CsA has antiviral effects on several viruses in different manners including herpes simplex virus (HSV), vaccinia virus (VV), BK polyomavirus (BKV), HIV-1 and hepatitis C virus (HCV) [3], [4], [7], [8], [16], [17], [18], [19], [20], [21]. Two well-studied antiviral effects of CsA was related to HCV and HIV [3], [4], [5], [6], [19], [20], [21], [22], [23]. As for HCV, CypB was firstly found to associate with NS5B and to stimulate its RNA binding activity and CsA primarily targeted NS5B by way of CypB to inhibit the viral replication [22], [24]. However, further studies indicated that CypA is crucial for HCV replication. CsA could inhibit the interaction between CypA and the NS5A protein of HCV and CsA also targeted the NS2 protein through CypA to inhibit the replication of HCV [3], [7], [8], [9]. Thus, CsA might combine with the both cyclophilin family proteins to regulate the replication of HCV. Upon HIV-1 infection, CsA disrupted the interaction between CypA and capsid (CA) and then inhibited the viral replication [4]. In addition, CsA could block the incorporation of HIV-1 envelope glycoprotein into virions [25]. In the present study, CsA was determined to inhibit the influenza A virus replication at the cell level. It was found that CsA enhanced the binding between CypA and M1 while not between CypB and M1. CsA treatment might increase the affinity of CypA to M1 to form ternary complex of CsA-CypA and M1. The formation of this complex might hinder the translocation of M1 protein during the replication of the virus. In addition, GST pull-down assay indicated that CypB interacted with M1 protein, while CsA had no effect on the binding ability between them. These results were in accordance with the previous study that over-expression or knock-down of CypB could not affect the influenza virus replication [11].

It was noticed that CsA treatment reduced the M1 protein expression level (Figure 3) but not the mRNA, vRNA and cRNA levels of M1 gene (Figure 4), suggesting that CsA perhaps inhibited influenza virus replication by targeting at a post-transcriptional level. The inhibitory abilities were different in 293T/CypA+ cell line compared with 293T/CypA− cell line (Figure 3). CsA inhibited the M1 protein level more effectively in 293T/CypA+ cell line than in 293T/CypA− cell line, which suggested CsA mainly targeted CypA protein for inhibiting the viral replication. However, there was still inhibitory effect of CsA on the influenza virus replication in 293T/CypA− cell line. Overall, the present evidence indicated that the effect of CsA on influenza A virus replication was not only through CypA− dependent pathway. There was a CypA− independent pathway to inhibit the replication of influenza A virus by targeting the post-transcription of influenza virus life cycle. The nuclear and cytoplasmic mRNA quantification of influenza viral mRNA indicated that CsA could impair the nuclear export of viral mRNA in the absence of CypA (Figure 5). As was reported, nuclear export of influenza A virus mRNAs required ongoing RNA polymerase II activity. Influenza A virus replication requires RNAP II activity not just to provide capped mRNA substrates but also to facilitate nuclear export of selected viral mRNAs of HA, M1 and NS1 genes while not NP and NEP [26]. CsA could inhibit the activity of RNA polymerase II while not RNA polymerase III or RNA polymerase I [27]. We deduced that the CypA-independent pathway of CsA was that CsA might inhibit the influenza virus replication through impairing the nuclear export of viral mRNA by inhibiting the activity of RNA polymerase II.

All currently approved anti-inﬂuenza drugs target essential viral functions and/or structures. The major drawback of this approach is that the virus will eventually adapt to the drug selective pressure [28]. To manipulate the interaction between host cell factor and virus which is essential for virus replication seems to be alternative way to develop the antiviral drug. In the present study, CsA inhibited the influenza virus replication partially through regulating functional CypA. In addition, the immunosuppression activity of CsA was not needed for its anti-influenza virus activity. So we can develop the derivatives of CsA which have low toxicity and high activity as the anti-influenza virus drug. In addition, CsA plays critical roles in the replications of several viruses through its high affinity with the cyclophilin family members. So CsA and its derivatives could be used as the promising strategies to explore the regulation mechanism of the virus replication.

## Materials and Methods

### Ethics statement

The use of all laboratory animals and animal subjects in our study was approved by the Beijing Association for Science and Technology, with approval ID SYXK (Beijing) 2007–0023, and all procedures were carried out in accordance with the Beijing Laboratory Animal Welfare and Ethical Guidelines of the Beijing Administration Committee of Laboratory Animals. Rabbit anti-CypA polyclonal antibody was prepared by Beijing Cowin Biotech Co., Ltd, Beijing, China, with approval from the Beijing Association for Science and Technology, ID SYXK (Beijing) 2007–0023. The authors supplied the purified His-CypA protein. The cell line HEK293T was generously provided by Jilong Chen, Institute of Microbiology, Chinese Academy of Sciences, who is also one of the authors of this manuscript.

### Cell lines, viruses and antibodies

Madin-Darby Canine Kidney (MDCK) cells (ATCC CCL-34), human embryonic kidney 293T cells [29] were maintained in Dulbecco's modified Eagle's medium (DMEM; GIBCO) supplemented with 10% heat-inactivated fetal bovine serum (FBS; GIBCO). Influenza A virus strain A/WSN/33 (H1N1) and A/Chicken/Liaoning/1/00 (H9N2) were used in these experiments, and they were propagated in MDCK cells. For rabbit anti-CypA polyclonal antibody, purified hexahistidine-tagged CypA (His- CypA) was provided to company (Beijing Cowin Biotech Co., Ltd, Beijing, China) for immunization. Mouse anti-M1 monoclonal antibody was prepared as described previously [30]. Mouse anti-NS1 monoclonal antibody (sc-130568) and anti-β-actin (sc-1616-R) were purchased from Santa Cruz Biotechnology.

### Construction of plasmids

The CypA and cyclophilin B (CypB) genes were obtained by PCR from the human kidney cDNA library (Clontech), and subcloned into the bacterial expression vector pGEX-6p-1, respectively. The full-length M1 gene of A/WSN/33 was subcloned into the bacterial expression vector pET30a (+) as described previously [11]. The full-length NP and M1 genes of PR8 were subcloned into the plasmid pCMV-Tag2, respectively.

### CPE assay

To study the effect of CsA (Sigma) on the cytopathic effect (CPE) in the influenza virus infected MDCK cells, 96-well microtiter plates were seeded with 10^4^ MDCK cells/0.1 ml per well. Monolayers were allowed to develop for 18 h in growth medium containing 10% fetal bovine serum at 37°C, resulting in 75% or greater confluency. The wells were washed with PBS and inoculated with 100 µl of influenza virus (A/WSN/33 and A/Chicken/Liaoning/1/00) diluted in DMEM with 2 µg/ml TPCK-treated trypsin (MOI = 0.1). After the virus was absorbed at 37°C for 1 h, the wells were washed with PBS, and then CsA was added at 2.5 µg/ml, 5 µg/ml, 10 µg/ml in DMEM with 2 µg/ml TPCK-treated trypsin, and then the preparations were incubated at 37°C for 36 h. The monolayers were stained with crystal violet (0.1% in 20% ethanol) and the CPE was examined.

### Growth curve

MDCK cells were inoculated with influenza virus (A/WSN/33) (MOI = 0.01) to determine the effect of CsA on the virus replication. Following adsorption for 1 h at 37°C, the virus-containing medium was removed and fresh medium was added to the cells in the absence or presence of CsA (5 µg/ml). At time points 0, 12, 24, 36, 48 h, the supernatants were collected and determined by the plaque titer on MDCK cells.

### RNA extraction and cDNA synthesis

Total RNA from transfected or infected cells were harvested by Trizol reagent (Invitrogen). Genomic DNA was digested by DNase Turbo (Ambion) for 30 min prior to reverse-transcription reactions. 1.5 mg of total RNA was reverse transcribed by SuperScript II reverse transcriptase (Invitrogen) and 10 pmol of vRNA specific primer (5′-AGCAAAGCAGG-3′; 5′-AGCAAAAGCAGG-3′) [31], 10 pmol of cRNA specific primer (5′-AGTAGAAACAAGG-3′) or 50 pmol of oligo-dT primer was used in each reaction. Negative controls (amplifications in the absence of RNA or primers) were included in parallel to ascertain absence of contamination by template nucleic acids and the efficiency in RT inactivation. The synthesized cDNA was subjected to real-time PCR analysis.

### Real-time PCR analysis

Real-time PCR was performed with the SYBR premix Ex taq (TaKaRa, Japan) on a corbett 6200 real time detection system (Corbett, Australia). The real-time PCR amplifications were carried out in triplicates in a total volume of 20 µl containing 10 µl SYBR green 2×premix, cDNA, primers (final concentration of 0.2 µM). The primers are as follows: the forward primer (5′-CCTGTCACCTCTGACTAAGGGG-3′) and the reverse primer (5′-TAGGGCATTTTGGACAAATCGT-3′), corresponding to nucleotide position between 153 and 252 of A/WSN/33 M1 gene [32]; The GAPDH mRNA served as an internal control using PCR primers: the forward primer of GAPDH (5′-GGTGGTCTCCTCTGACTTCAACA-3′) and the reverse primer of GAPDH (5′-GTTGCTGTAGCCAAATTCGTTGT-3′), as described in [33]. The PCR program was 95°C for 30 s followed by 40 cycles of 94°C for 5 s, 60°C for 30 s and dissociation curve analysis of amplification products was performed at the end of each PCR reaction to confirm that only one PCR product was amplified and detected. Each sample was run in triplicate along with the internal control gene. Dissociation curve analysis was performed after each assay to ensure specific target detection. Data analysis of real-time PCR was performed with Rotor Gene 6000 series Software (Corbett, Australia).

### Nuclear and cytoplasmic mRNA quantification by real-time PCR

Nuclear and cytoplasmic RNA was fractionated from cells as described in [12], [34]. Briefly, the cells were lysed in RSB (10 mM Tris, pH 7.4, 10 mM NaCl, 3 mM MgCl_2_) containing 0.5% Nonidet P-40, 10% glycerol, and 100 units/ml rRNasin (Promega). Nuclei were further washed with 1% Tween-40 and 0.5% sodium deoxycholate, and RNA from both cytoplasmic and nuclear fractions was purified using Trizol (Invitrogen). The purified RNA samples were treated with DNase I (TaKaRa) for 30 min at 37°C. One microgram of total RNA was used for cDNA synthesis with the oligo(dT)18 primer, followed by real-time RT-PCR quantification. Negative controls (amplifications in the absence of RNA or primers) were included in parallel to ascertain absence of contamination by template nucleic acids and the efficiency in RT inactivation. The standard plasmids for M1 and NP gene were constructed as reference standard used in absolute quantification assay. The resulting M1 and NP mRNA levels were detected by real-time quantitative PCR.

### Plaque assay

Plaque assays were performed as previously described [11]. MDCK cell monolayers (1.5×10^6^ cells at a confluency of 100% in 35 mm dishes) were washed with PBS^+^ (PBS supplemented with 0.5 mM MgCl_2_ and 1 mM CaCl_2_) and infected with different dilutions of virus for 1 h at 37°C. The virus inoculum was removed by washing with PBS. Cell monolayers were then overlaid with agar overlay medium (DMEM supplemented with 0.6% low-melting-point agarose and 2 µg/ml TPCK-treated trypsin) and incubated at 37°C. Visible plaques were counted at 2 d p.i. and the virus titers were determined. All data was expressed as the mean of three independent experiments.
